# Author Correction: Immunogenomic profiling determines responses to combined PARP and PD-1 inhibition in ovarian cancer

**DOI:** 10.1038/s41467-020-16344-z

**Published:** 2020-05-18

**Authors:** Anniina Färkkilä, Doga C. Gulhan, Julia Casado, Connor A. Jacobson, Huy Nguyen, Bose Kochupurakkal, Zoltan Maliga, Clarence Yapp, Yu-An Chen, Denis Schapiro, Yinghui Zhou, Julie R. Graham, Bruce J. Dezube, Pamela Munster, Sandro Santagata, Elizabeth Garcia, Scott Rodig, Ana Lako, Dipanjan Chowdhury, Geoffrey I. Shapiro, Ursula A. Matulonis, Peter J. Park, Sampsa Hautaniemi, Peter K. Sorger, Elizabeth M. Swisher, Alan D. D’Andrea, Panagiotis A. Konstantinopoulos

**Affiliations:** 1 Dana-Farber Cancer Institute, 450 Brookline Avenue, Boston, MA 02215 USA; 2 Harvard Medical School, 25 Shattuck Street, Boston, MA 02115 USA; 3 Research Program in Systems Oncology, University of Helsinki, Haartmaninkatu 8, 00014 Helsinki, Finland; 4 Laboratory of Systems Pharmacology, Harvard Medical School, Boston, 200 Longwood Avenue, MA 02115 USA; 5 TESARO: A GSK company, 1000 Winter Street, Waltham, MA 02451 USA; 6 Helen Diller Family Comprehensive Cancer Center, 1450 3rd Street, San Francisco, CA 94158 USA; 7 Brigham and Women’s Hospital, Laboratory for Systems Pharmacology, 75 Francis Street, Boston, MA 02115 USA; 8 Department of Pathology, Brigham and Women’s Hospital, Harvard Medical School, 75 Francis Street, Boston, MA 02115 USA; 9 Department of Biomedical Informatics, Harvard Medical School, 25 Shattuck Street, Boston, MA 02115 USA; 10 University of Washington, 1959 NE Pacific Street, Seattle, WA 98195 USA

**Keywords:** Cancer microenvironment, Immunotherapy, Translational research, Cancer, Cancer genomics

Correction to: *Nature Communications* 10.1038/s41467-020-15315-8, published online 19 March 2020.

The original version of this Article omitted the following from the Acknowledgements:

This research was also supported by the Ludwig Center at Harvard and U54-CA225088 to Peter Sorger, BioEntrepreneur-Fellowship of the University of Zurich, reference no. BIOEF-17-001 and Early Postdoc Mobility fellowship (no. P2ZHP3_181475) to Denis Schapiro.

The original version of this Article contained errors in the author affiliations.

Peter J. Park was incorrectly associated with Dana-Farber Cancer Institute, 450 Brookline Avenue, Boston, MA 02215, USA. The affiliation of Peter J. Park with Department of Biomedical Informatics, Harvard Medical School, 25 Shattuck Street, Boston, MA, 02115, USA was inadvertently omitted. This has now been corrected in both the PDF and HTML versions of the Article.

