# Supplementary material for: Immunogenomic profiling determines responses to combined PARP and PD-1 inhibition in ovarian cancer
Source: Nat Commun. 2020 Mar 19;11:1459. doi: 10.1038/s41467-020-15315-8 (PMC7081234; doi:10.1038/s41467-020-15315-8)

**Supplementary Materials**

**Immunogenomic profiling determines responses to combined PARP and PD-1 inhibition in ovarian cancer**

Farkkila et al,

Contents;

Supplementary Figure 1 - Distribution of BROCA and RAD51 assays and response, and validation of tumor mutational burden and Signature 3 inference method.

Supplementary Figure 2 - Chemotherapy exposure affects Nanostring gene expression analysis

Supplementary Figure 3 - Cell-type proportions in the tumor microenvironment

Supplementary Figure 4 - PD-L1 expression in the tumor microenvironment

Supplementary Table 5 – Multivariable Cox proportional hazards model for PFS

Supplementary Table 6 – Patient details of the two extreme responders

Supplementary File – Permutation test summary heatmap

Supplementary Figure 1

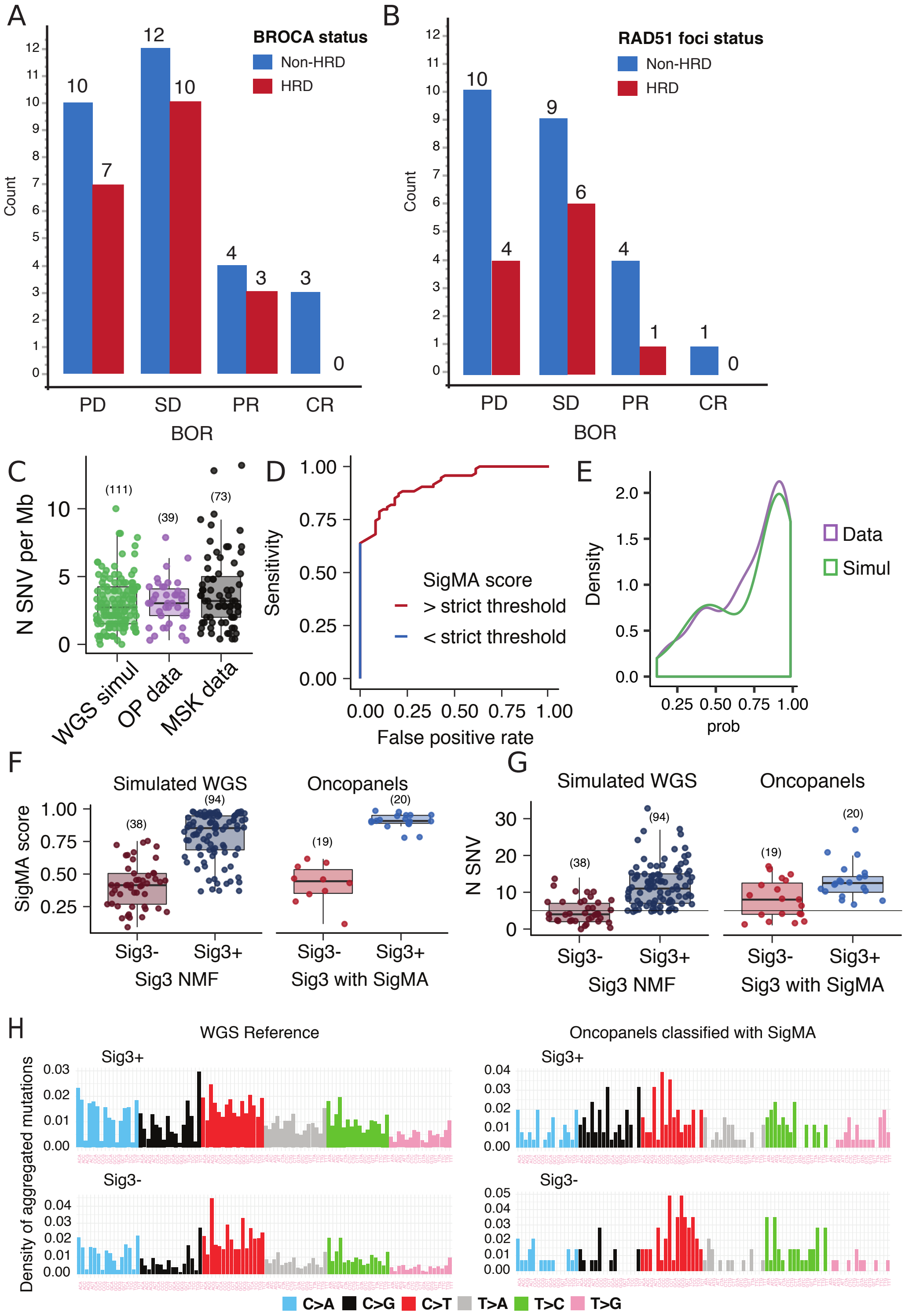

**Supplementary Figure 1 Distribution of BROCA and RAD51 assays and response, and validation of tumor mutational burden and Signature 3 inference method.**

A) Distributions of BROCA HRD status and B) RAD51 HRD and the best objective responses to niraparib and pembrolizumab. C) The number (nSNVs) per megabite (MB) compared in WGS, Oncopanel and a panel sequencing data. D) ROC curve showing sensitivity versus false positive rate determined using OncoPanel simulations from WGS data from ICGC (https://dcc.icgc.org/releases). The true positives and true negatives are determined based on presence of Signature 3 in WGS ovarian cancer data from ICGC consortium. The blue part of the ROC curve indicates SigMA scores below the strict threshold and the red part of the ROC curve indicates SigMA scores above the strict threshold corresponding to 2% false positive rate, and a sensitivity of 65%. **E)** Comparison of SigMA score for OncoPanel data from TOPACIO trial and OncoPanel simulations from WGS data showing that the simulations describe data well. **F)** (Left) Distribution of SigMA score in Sig3+ and Sig3- WGS samples, where Sig3 status is determined by a standard NMF based signature analysis from WGS data. (Right) Distribution of SigMA score in Sig3+ and Sig3- samples defined based on whether the SigMA score is above or below the strict threshold. **G)** Same as panel (c) but showing the total number of SNVs in four groups. **H)** (Top) The density of aggregated mutations from Sig3+ (Left) and Sig- (Right) samples in WGS data based on NMF based signature analysis (Middle) Same as the distributions on the top but for simulated OncoPanels that are classified into Sig3+ and Sig3- groups based on the SigMA score (Bottom) Same as the distributions in the middle but for OncoPanel data. On the right of each mutational spectra a bar graph shows the fraction of mutational signature exposures in each distribution, the colors indicate different signatures and Sig3 is shown in orange. Box plots are presented as the range (whiskers), center line as the median, and the bounds of box mark the highest and lowest quartiles. Number of samples is show in parenthesis.

# Supplemental Figure 2

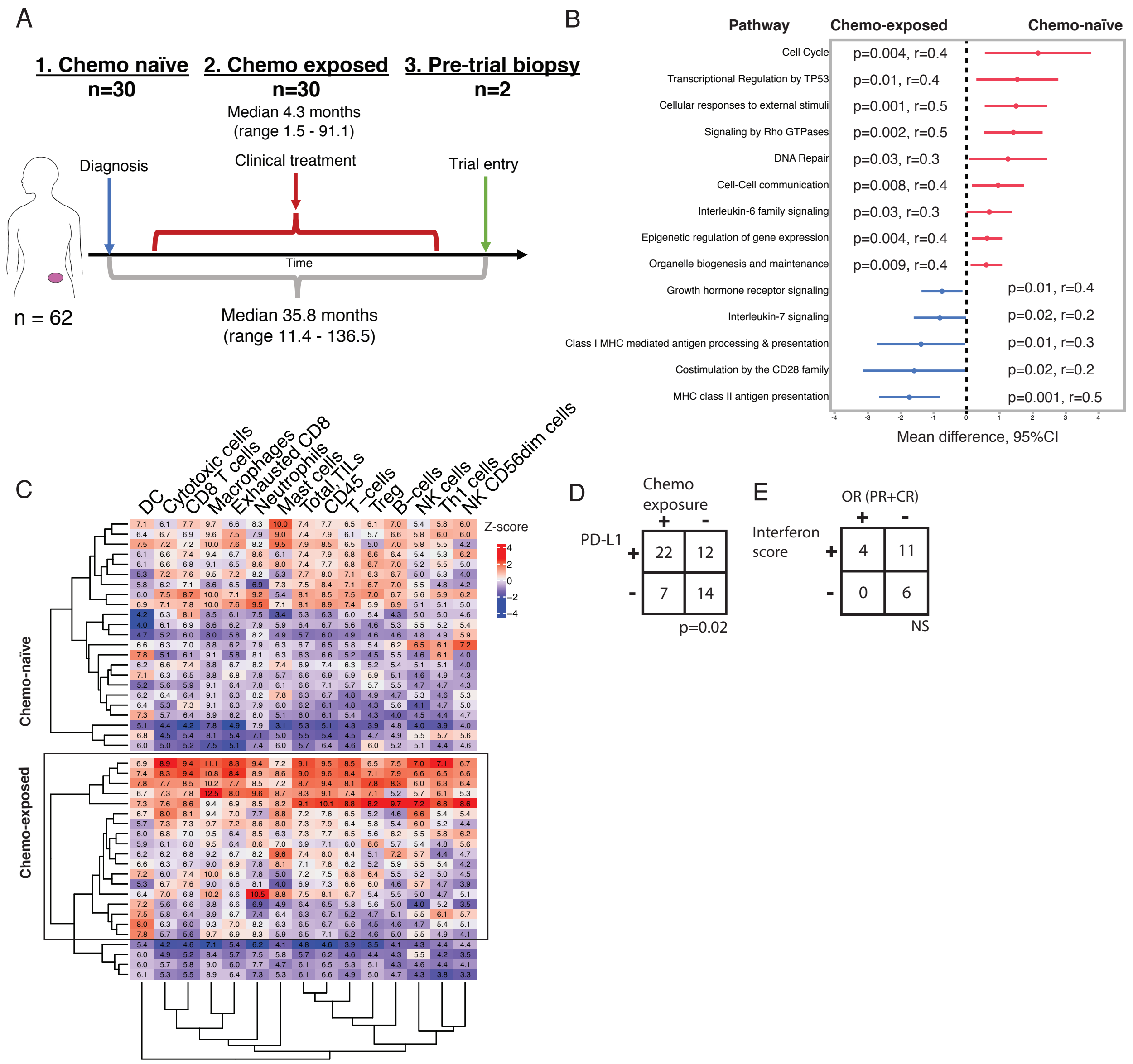

**Supplementary Figure 2 Chemotherapy exposure affects Nanostring gene expression analysis**
A) The pre-trial samples were collected either at diagnosis (chemo-naïve, n=30), during clinical treatment (chemo-exposed, n=30), or as pre-trial biopsies (n=2). B) In the samples available for Nanostring analysis, the chemo-exposure affected the gene expressions; the mean differences of the pathway scores between chemo-naïve (n=23) compared to chemo-exposed (n=22) are shown as dots, and lines represent the 95%CI. Pathways higher in chemo-naïve are displayed in red and pathways higher in chemo-exposed in blue (Mann-Whitney-U test; r = effect size). C) Chemo-exposed samples had higher z-scores for many of the immune cell types (black box). D) Chemo-exposure status significantly associated with PD-L1 immunohistohemistry (IHC) positivity measured as Complete Proportion Score ≥1 (Fisher's exact test). E) In chemo-naïve samples, all patients who responded were positive for interferon score as assigned by having the pathway score in the highest quartile of the range (≥75%) for any of the three Type-I interferon pathways (i.e. interferon score) (Fisher's exact test). All test were two-sided. No adjustment was made for multiple hypothesis testing (see materials and methods). Box plots are presented as the range (whiskers), center line as the median, bounds of box mark the highest and lowest quartiles, and the dashed line represents the mean.

## Supplementary Figure 3

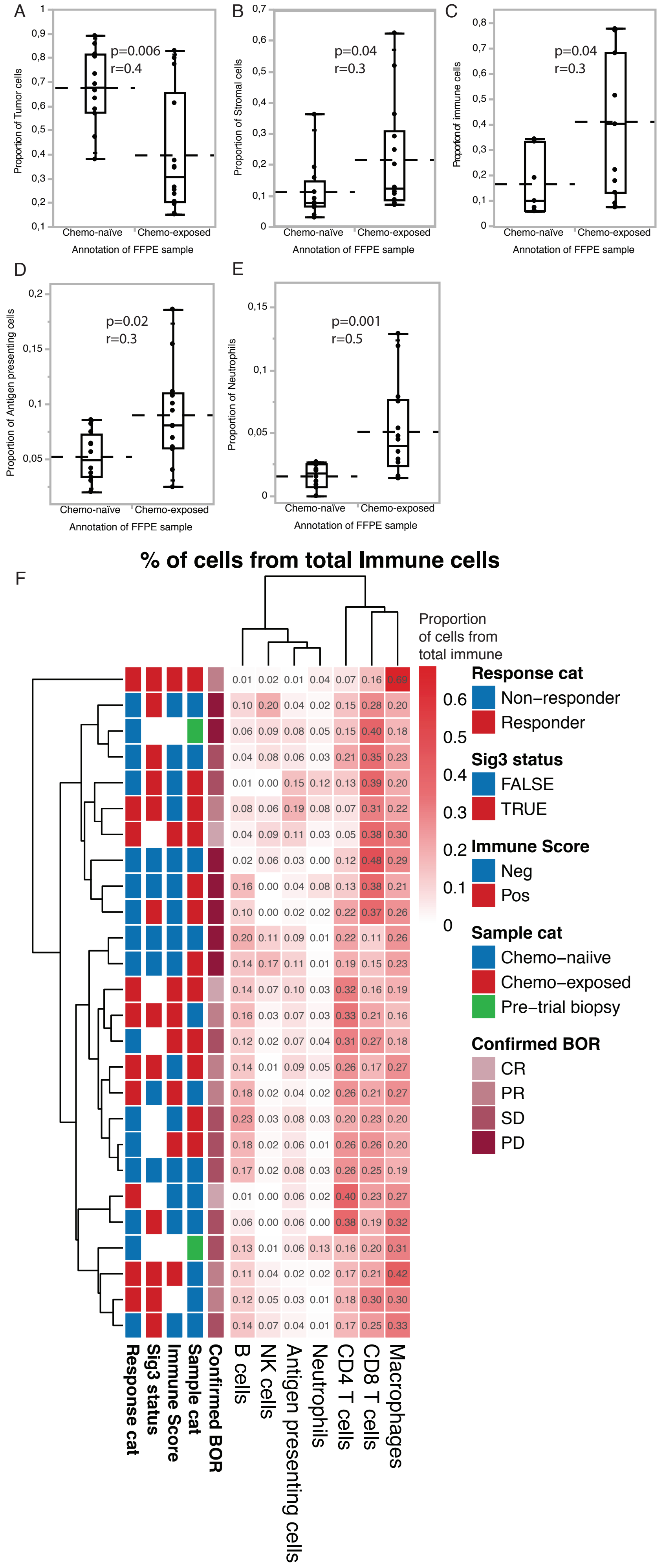

**Supplementary Figure 3 Cell-type proportions in the tumor microenvironment**

A) The chemo-exposed samples had lower proportion of tumor cells (Mann-Whitney-U), B) higher proportion of stromal (Mann-Whitney-U) and C) higher proportion of immune cells (Mann-Whitney-U) compared to the chemo-naïve samples. D) The chemo-exposed samples also had higher proportion of antigen presenting cells (Mann-Whitney-U), and E) neutrophils (Mann-Whitney-U) compared to chemo naïve samples. F) Heatmap of unsupervised hierarchical clustering of the proportions of immune cell subpopulations of the total immune cells (columns) and samples (rows) annotated by Sig3, Immune score, sample category and confirmed best objective response (BOR). All test were two-sided. No adjustment was made for multiple hypothesis testing. R= effect size. Box plots are presented as the range (whiskers), center line as the median, bounds of box mark the highest and lowest quartiles, and the dashed line represents the mean.

Supplementary Figure 4

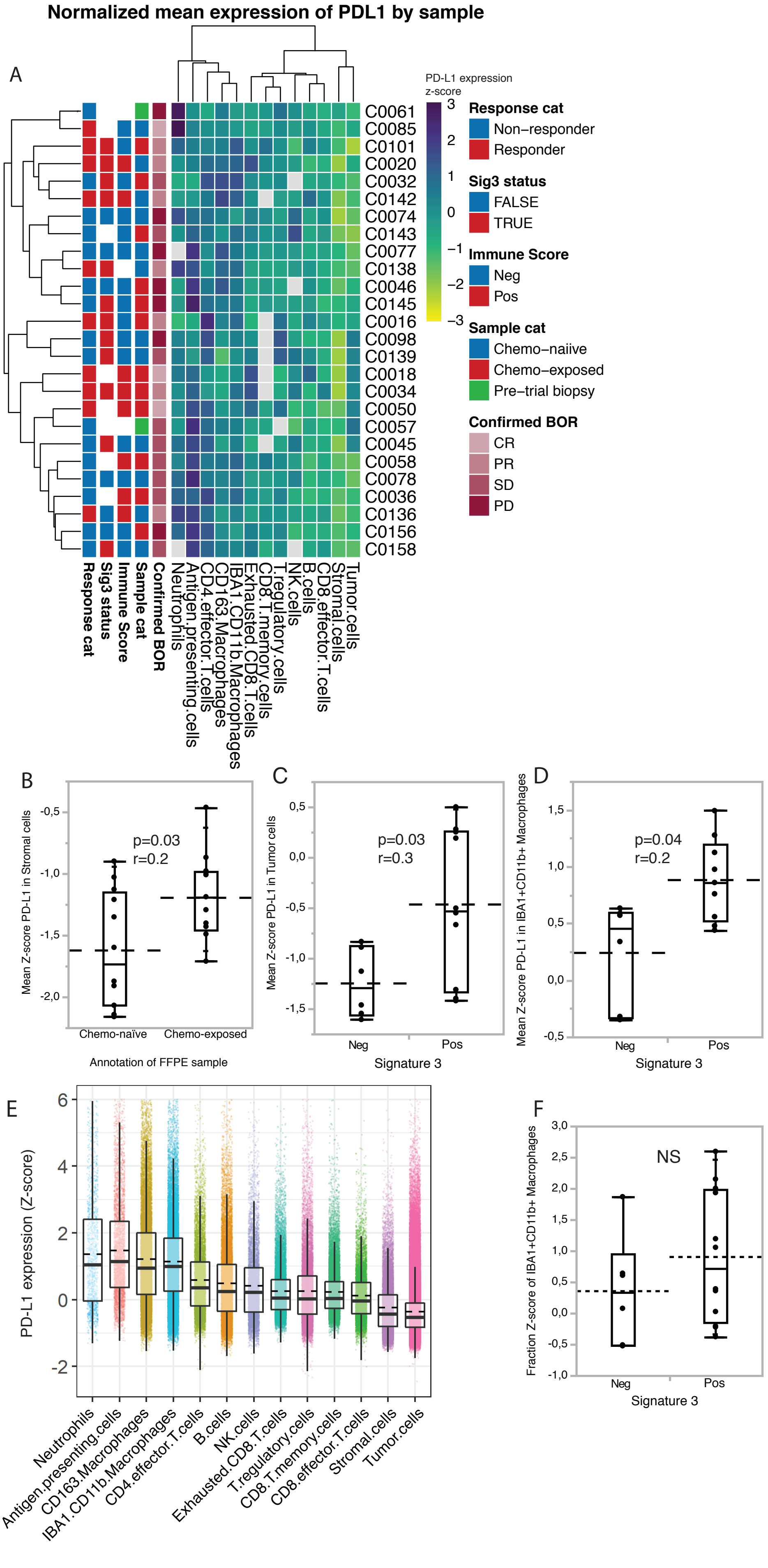

**Supplementary Figure 4 PD-L1 expression in the tumor microenvironment**

A) Heatmap of unsupervised hierarchical clustering of mean PD-L1 protein expression by cell types (columns) and samples (rows) annotated by Sig3, Immune score, sample category and confirmed best objective response (BOR). B) Mean PD-L1 in stromal cells was higher in the chemo-exposed compared to chemo-naïve samples Mann-Whitney-U). C) Mean PD-L1 expression was higher in the tumor cells (Mann-Whitney-U) and D) in IBA1+CD11b+ macrophages in Sig3 positive tumors Mann-Whitney-U). E) Normalized PD-L1 expression according to cell types in an extreme responder. Dots represent individual cells. F) Fraction (z-score) of PD-L1 positive macrophages neighboring exhausted CD8+T-cells and mutational Sig3 (Mann-Whitney-U). All test were two-sided. R= effect size. No adjustment was made for multiple hypothesis testing. Box plots are presented as the range (whiskers), center line as the median, bounds of box mark the highest and lowest quartiles, and the dashed line represents the mean.

Supplementary Table 5

Multivariable Cox proportional hazard model for progression-free survival.

| Factor | Risk Ratio | 95% CI | p-value |
|---|---|---|---|
| Age [1] | 1.09 | 0.20 – 5.7 | 0.92 |
| ECOG score [1] | 1.47 | 0.59 – 3.59 | 0.39 |
| Platinum sensitive [2] | 0.97 | 0.32 – 2.62 | 0.96 |
| PD-L1 IHC Positive | 0.95 | 0.35 – 2.16 | 0.76 |
| Immune Score Positive | 0.89 | 0.40 – 2.74 | 0.89 |
| Sig3 positive | 0.44 | 0.19 – 0.97 | 0.04* |

[1] at screening

[2] response to last platinum-based chemotherapy

Supplementary Table 6
Summary of the clinical and tumor characteristics of the two extreme responders.

| **Clinical details** | C0142 | C0138 |
|---|---|---|
| Age | 59 | 69 |
| ECOG at screening | 0 | 0 |
| Platinum sensitivity | Resistant | Resistant |
| Duration of treatment (days) | 378* | 358* |
| Best objective response (RECIST 1.1) | PR | PR |
| Duration of response (days) | 316* | 190* |
| Best tumor regression from baseline | -87% | -53% |
| **Tumor characteristics** | | |
| Tumor sample type | Chemo-naïve | Chemo-naïve |
| BRCA mutation | Wt | Wt |
| HRD test | Neg | Unk |
| BROCA HRD | Positive;<br>*BRCA1*<br>hypermethylation | Unk |
| RAD51 HRD | Neg | Unk |
| SigMA HRD | Positive | Positive |
| Immune score | Positive | Unk |
| Oncopanel | *TP53* c.560-1G>A | *TP53* c.578A>T<br>*BRIP1* c.3070G>A<br>*NF1* c.4207G>A<br>High copy number gain of *MYC*, *CD274* and *PDCD1LG2* |

* Ongoing at data cutoff

# Supplementary File; Neighbor to target significant attraction/ avoidance

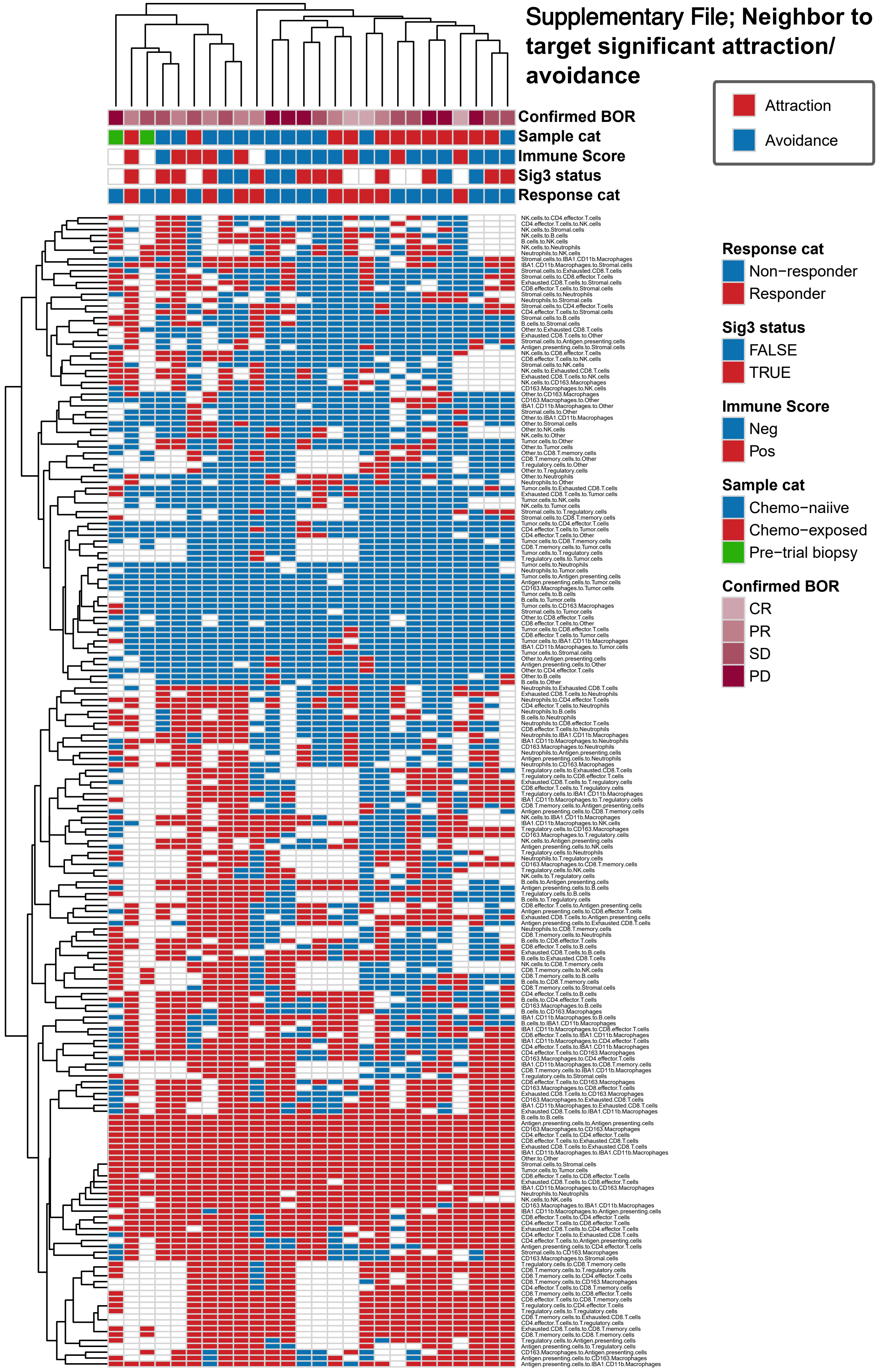

Supplement: Supplementary file 1 — Supplementary Information [file 41467_2020_15315_MOESM1_ESM.pdf]
