# Supplementary material for: Immunogenomic profiling determines responses to combined PARP and PD-1 inhibition in ovarian cancer
Source: Nat Commun. 2020 Mar 19;11:1459. doi: 10.1038/s41467-020-15315-8 (PMC7081234; doi:10.1038/s41467-020-15315-8)
Supplement: Supplementary file 4 — Reporting Summary [file 41467_2020_15315_MOESM4_ESM.pdf]

nature research

Corresponding author(s): Alan D'Andrea, Panagiotis Konstantinopoulos,
Last updated by author(s): Anniina Färkkilä

Reporting Summary

Nature Research wishes to improve the reproducibility of the work that we publish. This form provides structure for consistency and transparency in reporting. For further information on Nature Research policies, see Authors & Referees and the Editorial Policy Checklist .

Statistics

For all statistical analyses, confirm that the following items are present in the figure legend, table legend, main text, or Methods section.

n/a	Confirmed	
☐	☒	The exact sample size (*n*) for each experimental group/condition, given as a discrete number and unit of measurement
☐	☒	A statement on whether measurements were taken from distinct samples or whether the same sample was measured repeatedly
☐	☒	The statistical test(s) used AND whether they are one- or two-sided<br>*Only common tests should be described solely by name; describe more complex techniques in the Methods section.*
☐	☒	A description of all covariates tested
☐	☒	A description of any assumptions or corrections, such as tests of normality and adjustment for multiple comparisons
☐	☒	A full description of the statistical parameters including central tendency (e.g. means) or other basic estimates (e.g. regression coefficient) AND variation (e.g. standard deviation) or associated estimates of uncertainty (e.g. confidence intervals)
☐	☒	For null hypothesis testing, the test statistic (e.g. *F*, *t*, *r*) with confidence intervals, effect sizes, degrees of freedom and *P* value noted<br>*Give P values as exact values whenever suitable.*
☒	☐	For Bayesian analysis, information on the choice of priors and Markov chain Monte Carlo settings
☒	☐	For hierarchical and complex designs, identification of the appropriate level for tests and full reporting of outcomes
☐	☒	Estimates of effect sizes (e.g. Cohen's *d*, Pearson's *r*), indicating how they were calculated

Our web collection on statistics for biologists contains articles on many of the points above.

Software and code

Policy information about availability of computer code

Data collection	The following publicly available tools were used to collect the data;<br>SigMA Gulhan DC et al. Detecting the mutational signature of homologous recombination deficiency in clinical samples. Nat Genet. 2019;51(5):912-9.<br>BROCA; Walsh T, et al. Detection of inherited mutations for breast and ovarian cancer using genomic capture and massively parallel sequencing. Proc Natl Acad Sci U S A. 2010;107(28):12629-33<br>ASHLAR; (RRID:SCR_016266): Alignment by Simultaneous Harmonization of Layer/Adjacency Registration [Internet]. GitHub. 2018.<br>OncoPanel; Garcia EP, et al. Validation of OncoPanel: A Targeted Next-Generation Sequencing Assay for the Detection of Somatic Variants in Cancer. Arch Pathol Lab Med. 2017;141(6):751-8.
Data analysis	The following publicly available software were used to analyses the data;<br>HistoCat; Schapiro D et al. histoCAT: analysis of cell phenotypes and interactions in multiplex image cytometry data. Nat Methods. 2017;14(9):873-6.<br>NanoString https://www.nanostring.com/products/analysis-software/advanced-analysis<br>U-Net; Ronneberger O, et al. U-net: Convolutional networks for biomedical image segmentation. International Conference on Medical image computing2015.<br>FlowSOM; Lambrecht BN, et al. FlowSOM: Using self-organizing maps for visualization and interpretation of cytometry data. Cytometry A. 2015;87(7):636-45.<br>The code produced in this study can be accessed at; https://github.com/farkkilab/pubs/tree/master/Farkkila-et-al-1

For manuscripts utilizing custom algorithms or software that are central to the research but not yet described in published literature, software must be made available to editors/reviewers. We strongly encourage code deposition in a community repository (e.g. GitHub). See the Nature Research guidelines for submitting code & software for further information.

Data

Policy information about availability of data

All manuscripts must include a data availability statement. This statement should provide the following information, where applicable:

- Accession codes, unique identifiers, or web links for publicly available datasets
- A list of figures that have associated raw data
- A description of any restrictions on data availability

The data related to manuscript can be accessed at; DOI: 10.7303/syn21593960

Field-specific reporting

Please select the one below that is the best fit for your research. If you are not sure, read the appropriate sections before making your selection.

[x] Life sciences [ ] Behavioural & social sciences [ ] Ecological, evolutionary & environmental sciences

For a reference copy of the document with all sections, see nature.com/documents/nr-reporting-summary-flat.pdf

Life sciences study design

All studies must disclose on these points even when the disclosure is negative.

Sample size	The sample size was 62 patients recruited to the clinical trial. https://clinicaltrials.gov/ct2/show/NCT02657889
Data exclusions	Patients with no available data due to not enough tumor material were excluded from analyses.
Replication	There were no attempts for replication due to the nature of the study.
Randomization	There were no randomizations as the original study was a single-arm clinical trial.
Blinding	The investigators were blinded to group allocation at the time of data acquisition.

Reporting for specific materials, systems and methods

We require information from authors about some types of materials, experimental systems and methods used in many studies. Here, indicate whether each material, system or method listed is relevant to your study. If you are not sure if a list item applies to your research, read the appropriate section before selecting a response.

Materials & experimental systems

n/a	Involved in the study	
[ ]	[x]	Antibodies
[x]	[ ]	Eukaryotic cell lines
[x]	[ ]	Palaeontology
[x]	[ ]	Animals and other organisms
[ ]	[x]	Human research participants
[ ]	[x]	Clinical data

Methods

n/a	Involved in the study	
[x]	[ ]	ChIP-seq
[x]	[ ]	Flow cytometry
[x]	[ ]	MRI-based neuroimaging

Antibodies

Antibodies used	These are documented in supplementary table 2 of the manuscript.
Validation	Alla used antibodies have been internally validated with documentation shown in www.cycif.org.

Human research participants

Policy information about studies involving human research participants

Population characteristics	The patient population is described in detail in reference number 7, and supplementary Table 1 of the manuscript.
Recruitment	This explained in the reference number 7 of the manuscript and at https://clinicaltrials.gov/ct2/show/NCT02657889.
Ethics oversight	The study was conducted in accordance with ethical principles founded in the Declaration of Helsinki. This study received approval by the Dana-Farber Cancer Institute Institutional Review Board, and/or relevant competent authorities at each site.

Note that full information on the approval of the study protocol must also be provided in the manuscript.

Clinical data

Policy information about clinical studies

All manuscripts should comply with the ICMJEguidelines for publication of clinical research and a completedCONSORT checklist must be included with all submissions.

Clinical trial registration	NCT02657889
Study protocol	https://clinicaltrials.gov/ct2/show/NCT02657889
Data collection	This explained in the reference number 7 of the manuscript.
Outcomes	This explained in the reference number 7 of the manuscript. Outcomes of the clinical trial can be found at https://clinicaltrials.gov/ct2/show/NCT02657889
